# Data on the association between CTRP1 and future major adverse cardiovascular events in patients undergoing coronary angiography

**DOI:** 10.1016/j.dib.2019.104109

**Published:** 2019-06-07

**Authors:** Axel Muendlein, Andreas Leiherer, Christoph Saely, Janine Ebner, Kathrin Geiger, Eva Maria Brandtner, Alexander Vonbank, Peter Fraunberger, Heinz Drexel

**Affiliations:** a Vorarlberg Institute for Vascular Investigation and Treatment (VIVIT), Feldkirch, Austria; b Medical Central Laboratories, Feldkirch, Austria; c Private University of the Principality of Liechtenstein, Triesen, Liechtenstein; d Department of Medicine and Cardiology, Academic Teaching Hospital Feldkirch, Feldkirch, Austria; e Division of Angiology, Swiss Cardiovascular Center, University Hospital of Bern, Bern, Switzerland; f Drexel University College of Medicine, Philadelphia, PA, USA

**Keywords:** Major adverse cardiovascular events, CTRP1, Risk factor, Adipokine, Prospective study

## Abstract

This article provides additional data on the association of the new adipokine CTRP1 with the incidence of future major adverse cardiovascular events in a prospective cohort of patients undergoing coronary angiography. In this regard, multivariable Cox proportional hazards models taking into account cardiac risk markers are presented. Additionally, data on the impact of baseline variables including metabolic traits and co-morbidities on the incidence of future major adverse cardiovascular events are shown. This data article is associated to the research article titled ‘The Novel Adipokine CTRP1 is Significantly Associated with the Incidence of Major Adverse Cardiovascular Events’ Muendlein et al., 2019.

**Table:** Specifications table

Subject area	Medicine, Clinical Research
More specific subject area	Cardiology, Epidemiology, Biomarkers
Type of data	Table
How data was acquired	Serum CTRP1 levels were determined using a commercial enzyme-linked immunosorbent assay (ELISA) kit (Biovendor, Czech Republic) together with a DTX 880 Multimode Detector (Beckman Coulter, CA, U.S.A.). Patient data were acquired from national registries and review of patient registries. Hazard ratios were derived from univariable and multivariable Cox proportional hazards models using SPSS 25.0 for Windows.
Data format	Analyzed data
Experimental factors	Plasma CTRP1 levels were measured in 539 patients undergoing coronary angiography. These patients were prospectively followed, and major adverse cardiovascular events cardiovascular events were recorded for up to 8 years.
Experimental features	CTRP1 plasma concentrations were measured by ELISA
Data source location	Feldkirch, Austria
Data accessibility	Data is with this article. Raw data are made available for further investigations in collaborative studies.
Related research article	A. Muendlein, A. Leiherer, C.H. Saely, J. Ebner, K. Geiger, EM Brandtner, A. Vonbank, P. Fraunberger, H. Drexel, The Novel Adipokine CTRP1 is Significantly Associated with the Incidence of Major Adverse Cardiovascular Events, Atherosclerosis. 286 (2019). https://doi.org/10.1016/j.atherosclerosis.2019.04.222[1].

**Table:** 

**Value of the data** • The data presented here further characterize included patients by showing the impact of baseline variables on the incidence of major adverse cardiovascular events in patients undergoing coronary angiography, which may differ from the general population. • The data presented here stressed the impact of CTRP1 on future cardiovascular risk beyond its association with traditional coronary risk factors and, therefore, may stimulate further research on the role of CTRP1 in the development of cardiovascular disease. • These data are important, because the existing literature about CTRP1 is still limited, and therefore, they particularly extend the knowledge regarding the association between CTRP1 and cardiovascular disease.

## 1 Data

A significant association between CTRP1 and major adverse cardiovascular events (MACE) in patients undergoing coronary angiography has been reported in the associated research article [1]. In addition to the baseline characteristics of the selected study population given in the main article, Table 1 of this article shows the impact of most baseline variables on the incidence of MACE. Table 2 shows further data regarding multivariable Cox proportional hazards models adjusting for age, sex, body mass index, type 2 diabetes mellitus, significant coronary artery disease, hypertension, smoking, LDL cholesterol, HDL cholesterol, and estimated glomerular filtration rate and additionally for the extent of coronary artery disease as well as the percentage of left ventricular ejection fraction (model a) or inflammatory markers including fibrinogen and C-reactive protein (model b).

**Table 1:** Impact of baseline variables on the incidence of major adverse cardiovascular events.

	HR (95%CI)	p-value
Age (years)	1.20 [0.96–1.49]	0.116
Male gender	0.99 [0.68–1.43]	0.945
BMI (kg/m^2^)	0.86 [0.72–1.04]	0.862
Metabolic syndrome	1.33 [0.94–1.88]	0.111
Type 2 diabetes mellitus	1.76 [1.23–2.50]	0.002
Hypertension	1.12 [0.72–1.77]	0.613
Smoking	1.21 [0.84–1.74 ]	0.302
LDL-Cholesterol (mg/dl)	0.84 [0.71–1.00]	0.049
HDL-Cholesterol (mg/dl)	0.74 [0.62–0.90]	0.002
Triglycerides (mg/dl)	1.06 [0.88–1.26]	0.550
eGFR (ml/min/1.73m^2^)	0.70 [0.58–0.85]	<0.001
NAFLD	1.14 [0.79–1.65]	0.482
Significant CAD	1.65 [1.12–2.44]	0.012
Extent of CAD	1.15 [1.05–1.26]	0.002
LVEF (%)	0.61 [0.51–0.73]	<0.001
C-reactive protein (mg/dl)	1.46 [1.23–1.73]	<0.001
Fibrinogen (mg/dl)	1.22 [1.01–1.47]	0.036
BNP (pg/dl)	1.51 [1.31–1.75]	<0.001

Results of univariate Cox regression analysis. Age, BMI, LDL-C, HDL-C, triglycerides, eGFR, LVEF, C-reactive protein, fibrinogen, and BNP were log-transformed and z-transformed before analysis. Coronary artery stenoses with stenotic narrowing ≥50% were defined as significant CAD. The extent of CAD was defined as the number of ≥50% lesions. BMI, body mass index; eGFR, estimated glomerular filtration rate; NAFLD, non-alcoholic fatty liver disease; CAD, coronary artery disease; LVEF, left ventricular ejection fraction; BNP, brain natriuretic peptide; HR, hazard ratio; CI, confidence interval.

**Table 2:** Adjusted associations between CTRP1 quartiles and the incidence of major adverse cardiovascular events - results from multivariable Cox regression analyses.

	CTRP1 quartiles				
	Quartile 1	Quartile 2	Quartile 3	Quartile 4	p_trend_-value
Adjusted^a^ hazard ratio (95% CI)	1^reference^	1.98 [1.12–3.50]; p = 0.019	2.18 [1.23–3.77]; p = 0.005	1.86 [1.06–3.27]; p = 0.030	0.036
Adjusted^b^ hazard ratio (95% CI)	1^reference^	1.83 [1.04–3.23]; p = 0.037	2.16 [1.25–3.75]; p = 0.006	1.80 [1.03–3.15]; p = 0.038	0.041

Adjustment model ^a^ adjusts for age, sex, BMI, type 2 diabetes mellitus (T2DM), angiographically significant coronary artery disease (CAD), the extent of CAD, percentage of left ventricular ejection fraction (LVEF), hypertension, smoking, LDL cholesterol, HDL cholesterol, and estimated glomerular filtration rate (eGFR); model ^b^ adjusts for age, sex, BMI, type 2 diabetes mellitus (T2DM), angiographically significant coronary artery disease (CAD), hypertension, smoking, LDL cholesterol, HDL cholesterol, estimated glomerular filtration rate, (eGFR), fibrinogen, and C-reactive protein (CRP). Age, BMI, LVEF, LDL cholesterol, HDL cholesterol, eGFR, fibrinogen, and CRP were log-transformed before included in multivariable Cox regression analyses.

## 2 Experimental design, materials and methods

The present dataset included 539 consecutive Caucasian patients, who were referred to elective coronary angiography for the evaluation of established or suspected stable CAD at the academic teaching hospital Feldkirch, Austria. Baseline characteristics were obtained as described in the associated main article [1] and in previous reports [2], [3]. In short, the extent of atherosclerosis was defined as the number of ≥50% lesions. Left ventricular function was assessed by 2D echocardiography. Venous blood samples were collected after an overnight fast of 12 h prior to angiography and laboratory measurements were performed from fresh serum or plasma samples or from serum or plasma samples stored at −80 °C. C-reactive protein (CRP) was measured by particle enhanced immunological agglutination (Roche, Switzerland) on a Hitachi Cobas 501. Serum CTRP1 levels were determined using a commercial enzyme-linked immunosorbent assay (ELISA) kit (Biovendor, Brno, Czech Republic; article number: RD191153100R).

During a mean follow-up period of 5.9 ± 2.2 years (with a total of 8 years) cardiovascular events were recorded. Out of the 539 patients initially included in the present study, 15 subjects were lost to follow-up. MACE was defined as a three-point composite of cardiovascular death, nonfatal myocardial infarction, and nonfatal stroke. Time and causes of death were regularly obtained from a national survey (Statistik Austria, Vienna, Austria) or from hospital records.

Hazard ratios (HRs) and 95% confidence intervals of the HRs were derived from univariable and multivariable Cox proportional hazards models; log-transformed continuous variables were z-transformed for these analyses. P-values <0.05 were considered significant. Statistical analyses were performed with SPSS 25.0 for Windows (IBM, Armonk, New York, USA).

The present study has been approved by the Ethics Committee of the University of Innsbruck, Austria, and written informed consent was given by all participants.

## References

[1] Muendlein A., Leiherer A., Saely C.H., Ebner J., Geiger K., Brandtner E.M., Vonbank A., Fraunberger P., Drexel H. (2019). The Novel adipokine CTRP1 is significantly associated with the incidence of major adverse cardiovascular events. Atherosclerosis.

[2] Muendlein A., Saely C.H., Leiherer A., Fraunberger P., Kinz E., Rein P., Vonbank A., Zanolin D., Malin C., Drexel H. (2014). Angiopoietin-like protein 4 significantly predicts future cardiovascular events in coronary patients. Atherosclerosis.

[3] Leiherer A., Muendlein A., Saely C.H., Ebner J., Brandtner E.M., Fraunberger P., Drexel H. (2017). Serum uromodulin is a predictive biomarker for cardiovascular events and overall mortality in coronary patients. Int. J. Cardiol..

